# Microcatheter-Directed Thrombolysis Using Recombinant Tissue Plasminogen Activator for the Treatment of Acute Superior Mesenteric Artery Embolism: A Case Report

**DOI:** 10.3390/medicina59111889

**Published:** 2023-10-24

**Authors:** Yang-Won Kim, Ho-Cheol Choi, Won-Jeong Yang, Byeong-Ju Koo, Jae-Kyeong Ahn, Jeong-Pyo Lee, Jae-Bum Na, Sa-Hong Jo, Sung-Eun Park, Jung-Ho Won

**Affiliations:** 1 Department of Radiology, Gyeongsang National University School of Medicine and Gyeongsang National University Hospital, Jinju 52727, Republic of Korea; rladiddnjs89@gnuh.co.kr (Y.-W.K.); hocheol72@gnuh.co.kr (H.-C.C.); yangwonjeong@gnuh.co.kr (W.-J.Y.); tyfmfgofk@gnuh.co.kr (B.-J.K.); jakyong28@gnuh.co.kr (J.-K.A.); cholsoo12@gnuh.co.kr (J.-P.L.); jbna@gnu.ac.kr (J.-B.N.); 2 Department of Radiology, Gyeongsang National University School of Medicine and Gyeongsang National University Changwon Hospital, Changwon 51472, Republic of Korea; jo452y@gnuh.co.kr (S.-H.J.); uneyes@gnuh.co.kr (S.-E.P.)

**Keywords:** acute mesenteric ischemia, embolectomy, endovascular treatment, revascularization, superior mesenteric artery, thrombolysis

## Abstract

*Background*: Acute mesenteric ischemia (AMI) is a life-threatening condition, and in 50% of patients, AMI is caused by acute superior mesenteric artery (SMA) embolism. Endovascular treatment is increasingly being considered the primary modality in selected cases. Many studies have reported that percutaneous aspiration embolectomy using a guiding catheter and thrombolysis with recombinant tissue plasminogen activator (rtPA) are effective in treating SMA embolism. However, no reports on treating SMA embolism using rtPA administered via a microcatheter exist. *Case presentation*: A 64-year-old man with underlying atrial fibrillation presented with acute SMA embolism revealed using computed tomography (CT). rtPA (total 3 mg) was carefully administered into the occluded SMA through a microcatheter. No complications occurred, and complete revascularization of the SMA was revealed on follow-up CT. *Conclusions*: Compared with previous reports, this case report reveals that successful revascularization can be achieved using rtPA administered via a microcatheter, with a low dose of rtPA and a short duration of thrombolysis.

## 1. Introduction

Acute mesenteric ischemia (AMI) is a rare but life-threatening disease that is caused by sudden disruption of blood flow to the bowels. AMI includes mesenteric arterial embolism, mesenteric arterial thrombosis, mesenteric venous thrombosis, and non-occlusive mesenteric ischemia (NOMI) [1]. Among them, mesenteric arterial embolism accounts for approximately 50% of AMI cases, and a significant proportion of these emboli are caused by atrial fibrillation [2].

A delayed diagnosis of AMI can result in bowel necrosis or perforation, and early diagnosis and treatment are essential for a better prognosis. Physical examination may demonstrate nonspecific findings; however, if it reveals signs of peritoneal irritation, an increased possibility of bowel ischemia or necrosis exists [3].

Duplex sonography has limited value in detecting emboli beyond the proximal main vessel or NOMI, and therefore mesenteric angiography has been considered the gold standard for diagnosing AMI [4].

Recently, computed tomography angiography (CTA) has become a diagnostic modality for AMI. CTA not only enables combined vascular and bowel assessment but also helps in differentiating between occlusive and non-occlusive causes.

Previously, open surgery was the only treatment modality for AMI. Currently, endovascular treatment (EVT) has been increasingly used as an alternative treatment option and is associated with low mortality compared with open surgery [5]. If the patient shows no signs of peritoneal irritation, the disease progression is within 12 h, and an embolus is confirmed on CTA, endovascular thrombectomy or thrombolysis can be considered a treatment option [6].

When there is acute angulation between the superior mesenteric artery (SMA) and the aorta, advancing the aspiration catheter into the SMA can be problematic. Additionally, endovascular thrombectomy and thrombolysis are associated with complications, including dissection of the SMA and gastrointestinal bleeding [7,8].

To the best of our knowledge, no case of using a microcatheter for thrombolysis using recombinant tissue plasminogen activator (rtPA) for treating SMA embolism has been reported. Herein, we report a case of successful microcatheter-directed thrombolysis using rtPA in a patient with SMA embolism.

## 2. Detailed Case Description

A 64-year-old man presented to our emergency department with epigastric pain, vomiting, and diarrhea. He was diagnosed with atrial fibrillation 30 years ago but did not undergo any medical treatment.

Laboratory tests showed leukocytosis (17.8 × 10^3^/mm^3^); other laboratory findings, including lactic acid levels, were normal. Physical examination revealed abdominal tenderness but no signs of peritoneal irritation.

Abdominal examination revealed nonspecific findings without any signs of ileus or free air. Contrast-enhanced computed tomography (CT) was performed to evaluate the cause of abdominal pain.

**Figure 1:**
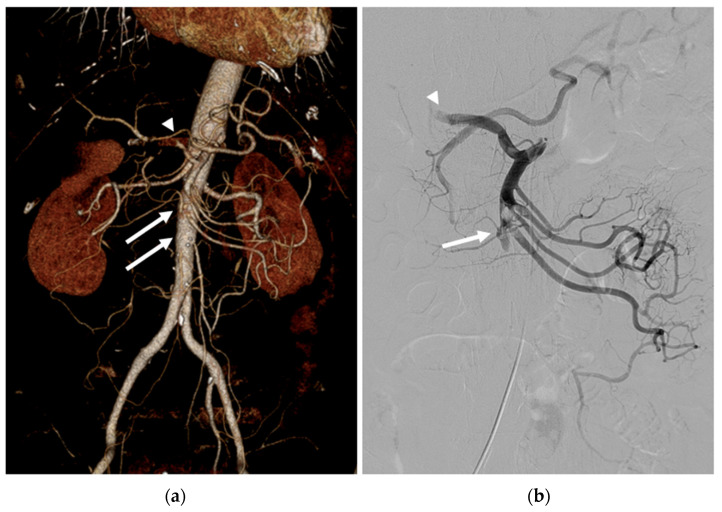
(**a**) Three-dimensional volume-rendered image of abdominal CT angiography revealed a segmental thrombus in the proximal SMA (arrows) and a focal thrombus in the CHA (arrowhead). A hepaticomesenteric common trunk was also observed. (**b**) Initial angiography revealed occlusion of the proximal SMA (arrow) and the CHA (arrowhead).

The CT revealed a segmental thrombus in the SMA, a focal thrombus in the common hepatic artery (CHA), and splenic infarction. A hepaticomesenteric common trunk, a rare anatomical variant of the hepatic artery, was observed. (Figure 1a) Moreover, no signs of peritonitis or bowel ischemia were observed. Because his symptoms started three hours previously without peritoneal irritation, the emergency physician referred the patient to us for EVT.

A 6F sheath was introduced into the right common femoral artery, and the SMA was selectively catheterized using a 5 Fr RH catheter (Cook Medical, Bloomington, IN, USA). Angiography revealed proximal SMA and CHA occlusions (Figure 1b).

We exchanged a 5 Fr RH catheter over a 0.035″ guidewire (Terumo, Tokyo, Japan) with a 6 Fr guiding catheter (Flexor Check-Flo introducer, Cook Medical, Bloomington, IN, USA) but failed to advance the guiding catheter into the proximal SMA due to the acute angle between the SMA and aorta.

**Figure 2:**
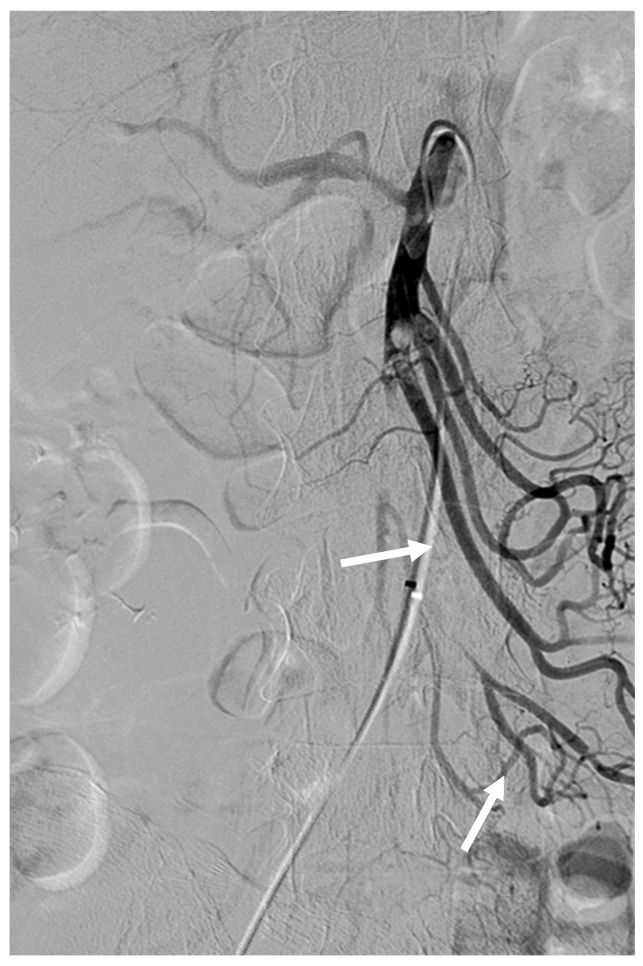
After thrombolysis using 2 mg of rtPA via RH catheter, angiography revealed partial recanalization of jejunal and ileal branches of the SMA (arrows).

After several attempts, we decided to change our plan of performing embolothrombectomy to thrombolysis using a 5 Fr RH catheter and microcatheter (Progreat Lambda, Terumo, Somerset, NJ, USA) with rtPA. We diluted 5 mg of rtPA in 25 mL of saline and 25 mL of contrast solution to prepare an rtPA solution. Subsequently, the SMA was selectively catheterized using a 5 Fr RH catheter. rtPA (2 mg; 20 mL of rtPA solution) was carefully injected (1 mg per 10 min) through the RH catheter. After thrombolysis, angiography revealed partial recanalization of the proximal SMA and its jejunal and ileal branches (Figure 2). Segmental SMA occlusion with sluggish jejunal flow remained, and no visible colic flow was observed.

**Figure 3:**
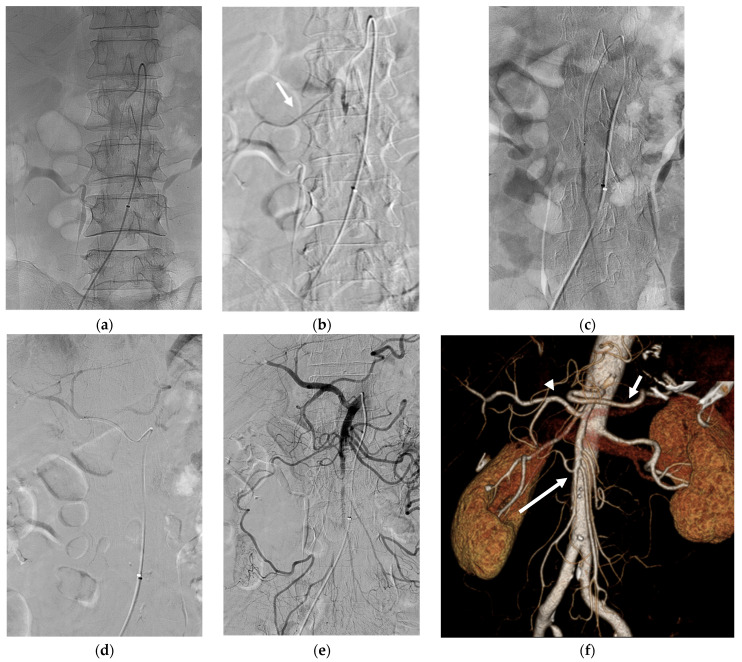
(**a**) The 1.9Fr microcatheter was advanced in front of the SMA occlusion site and 0.5 mg of rtPA was injected for 10 min. (**b**) After the injection, angiography revealed partial recanalization of right colic flow (arrow). (**c**) The microcatheter was then advanced into the middle and distal SMA and 0.5 mg of rtPA was injected for 10 min. (**d**) The CHA was also selectively catheterized using the microcatheter and 0.5 mg of rtPA was injected for 10 min. (**e**) Final angiography revealed near total recanalization of SMA and its branches. (**f**) After 2 days, follow-up three-dimensional volume-rendered image of abdominal CT angiography revealed successful recanalization of the SMA (long arrow), the CHA (arrowhead), and the splenic artery (short arrow).

A 1.9 Fr microcatheter was advanced immediately in front of the SMA occlusion site, and 0.5 mg of rtPA (5 mL solution) was injected for 10 min. After the injection, angiography revealed partial recanalization of right colic flow. The microcatheter was then advanced into the middle and distal branches of the SMA, and rtPA (0.5 mg) was injected again for 10 min. Additionally, the CHA and splenic artery were selectively catheterized using a 5 Fr RH catheter and a microcatheter. Subsequently, 0.5 mg of rtPA was injected into each artery in front of the occluded site. The final angiogram revealed near-complete revascularization of the SMA (Figure 3). To avoid bleeding complications, no additional rtPA was administered.

After the procedure, the patient was admitted to the intensive care unit and was administered continuous intravenous heparin infusion. No immediate major or minor postoperative complications occurred. After two days, contrast-enhanced CT revealed successful recanalization of the SMA and CHA occlusion without bowel ischemia or active bleeding. Eight days after thrombolysis, the patient was discharged and subsequently treated with warfarin for atrial fibrillation.

## 3. Discussion

Currently, EVT is increasingly being used for acute SMA occlusion in patients without peritonitis or bowel ischemia. EVT includes percutaneous aspiration thrombectomy, thrombolysis, angioplasty, and stenting.

Several studies have reported that percutaneous aspiration embolectomy with a guiding catheter is useful for treating SMA occlusion [9,10]. For aspiration, a guiding catheter (minimum diameter, 6 Fr) should be placed in the SMA. However, similar to the present case, challenges in advancing the guiding catheter to a stable position in the main stem of the SMA can occur owing to the acute angle between the SMA and the abdominal aorta or aortic tortuosity, especially for less experienced interventionists. Once the SMA is catheterized using a 5 Fr catheter, advancing the microcatheter to the SMA is technically less challenging than advancing the guiding catheter, even for less experienced interventionists.

A percutaneous brachial artery approach could be an alternative option to overcome the acute angulation of the SMA. A minimum 6 Fr sheath is required for aspiration embolectomy. In a study, the complication rate of brachial access was higher (10.6%) than that of femoral access (1.4–3.7%), with an increased risk of complications (e.g., hematoma, median neuropathy, bleeding, or thrombus) associated with increasing sheath size [11]. Moreover, additional puncture of the brachial artery may prolong the procedure time. Therefore, femoral access may be a better choice for these patients.

Thrombolysis with rtPA or urokinase is also an effective treatment option for acute SMA occlusion [12,13]. To the best of our knowledge, no study has compared the effectiveness and safety of low-dose rtPA and urokinase in the treatment of acute SMA occlusion. Sugimoto et al. reported that rtPA and urokinase were equally effective and safe agents for catheter-directed thrombolysis in patients with peripheral arterial occlusive disease and deep-vein thrombosis. They also reported that thrombolysis with rtPA had a significantly shorter duration than that with urokinase (rtPA: 24.6 ± 11.2 h, urokinase: 33.3 ± 13.3 h) [14]. Considering the duration of thrombolysis, we chose rtPA over urokinase as the thrombolytic agent.

Björnsson et al. reported a successful thrombolysis rate of 88% (30/34 patients) with rtPA treatment for acute SMA occlusion. There were six cases of self-limiting bleeding complications. The median dose of rtPA was 20 mg (interquartile range [IQR], 11.6–34.0 mg), and the median duration of thrombolysis was 22 h (IQR, 10.0–30.5 h) [12].

In the present case, 2 mg of rtPA (1 mg every 10 min) was slowly and carefully injected into the proximal SMA via a 5 Fr RH catheter. After achieving partial recanalization, the microcatheter was advanced into the proximal SMA, and rtPA (0.5 mg) was injected for 10 min. Subsequently, the microcatheter was advanced into the distal SMA branch in close proximity to the residual thrombus to prevent the injection of rtPA into the recanalized side branches of the SMA. An additional 0.5 mg of rtPA was injected slowly into the distal SMA for 10 min. Only 3 mg of rtPA was injected into the SMA (total 4 mg, including the CHA and splenic artery), and the duration of thrombolysis was 58 min. Compared to the study by Björnsson et al. (2011) [12], the dose of rtPA and the duration of thrombolysis were considerably lower in the present case. Furthermore, no complications occurred during or immediately after the procedure.

The present case demonstrated that successful thrombolysis could be achieved within a short duration, even with ultra-low-dose rtPA. Using a microcatheter allows better control and selective administration of rtPA to occluded branches, minimizing the risk of unwanted administration through normal or recanalized side branches. Therefore, microcatheter-directed thrombolysis in close proximity to the occlusion site helps reduce the potential risk of complications such as gastrointestinal bleeding or systemic hemorrhage. Additionally, decreased duration of thrombolysis may reduce the patient’s discomfort caused by the continuous infusion of rtPA for several hours and potentially shorten the hospitalization period.

## 4. Conclusions

Successful recanalization was achieved in the present case of acute SMA occlusion. Microcatheter-directed thrombolysis with rtPA can reduce the dose of rtPA and the duration of the procedure. Thus, it may be a safe and effective treatment option for acute SMA embolism.

## Data Availability

Not applicable.
